# Same ICD-10 code, different estimands: four Polish National Health Fund obesity series, 2015–2025

**DOI:** 10.3389/fpubh.2026.1920632

**Published:** 2026-09-18

**Authors:** Marcin Poboży, Anna Rulkiewicz, Wojciech Lisik, Zoran Stojčev, Ewa Filipiak-Rokicka, Iwona Pilchowska, Justyna Domienik-Karłowicz

**Affiliations:** 1 NZOZ Cichowski Pobozy Healthcare Facility, Maciejowice, Poland; 2 LUX MED Sp. z o.o., Warsaw, Poland; 3 Department of General, Oncological, Metabolic and Thoracic Surgery, Military Institute of Medicine – National Research Institute, Warsaw, Poland; 4 2nd Department of General, Vascular and Oncological Surgery, Faculty of Medicine and Dentistry, Czerniakowski Hospital, Medical University of Warsaw, Warsaw, Poland; 5 University of Medical Sciences in Warsaw, Warsaw, Poland; 6 Department of Internal Medicine and Cardiology with the Centre for Diagnosis and Treatment of Venous Thromboembolism, Infant Jesus Clinical Hospital, University Clinical Centre, Medical University of Warsaw, Warsaw, Poland

**Keywords:** administrative data, ecological time series, ICD-10, National Health Fund, obesity, Poland, routinely collected health data

## Abstract

**Background:**

Poland's National Health Fund (NFZ) publishes four national series indexed by ICD-10 code E66. Although their labels suggest related measures of obesity, differences in analytical unit, entry rule, look-back period, and temporal structure may alter both the reported level and the apparent response to healthcare-system disruption. We examined whether selecting an official NFZ E66 series materially changes the level, trend, and permissible public-health interpretation of recorded obesity-related activity.

**Methods:**

This retrospective, descriptive ecological time-series study audited 44 annual resources covering registered morbidity, first-recorded patients, annual E66 patients, and E66-coded services in 2015–2025. We analyzed counts and population-scaled administrative rates, summarized long-term change using log-linear annual percentage change (APC), and used a one-year 2020 indicator as a descriptive pandemic-era stress test. An endpoint sensitivity analysis excluded 2025. Independent BMI sources provided cross-sectional contextual triangulation for 2025.

**Results:**

In 2025, two official person-based series yielded 1,534,022 persons with a current or earlier principal E66 record and 325,769 persons with qualifying current-year E66 activity, a 4.7-fold difference arising from construction rather than alternative estimates of a single quantity. From 2015 to 2025, endpoint increases ranged from 178.1 to 332.5%, and population-scaled APCs from 10.5 to 14.5%. During 2020, the first year of the COVID-19 pandemic, deviations from the fitted trend were modest for registered morbidity (−3.5%) but substantially larger for the three series requiring current-year activity (−39.4 to −44.3%).

**Conclusions:**

The same E66 code supports four distinct administrative trajectories. Their growth may reflect a genuine rise in clinical obesity together with changes in recognition, access, recording, and service delivery; the aggregates cannot separate these contributions. Explicit estimand definitions are therefore essential when NFZ indicators are used for surveillance or policy.

## Introduction

Obesity is a chronic, complex, systemic disorder characterized by excess, abnormal distribution, or dysfunction of adipose tissue that impairs health. While body mass index (BMI) remains a useful tool for population monitoring, individual clinical evaluation should also consider adiposity distribution, organ impairment, and functional limitations (1). Obesity is linked to dyslipidaemia, type 2 diabetes, arterial hypertension, sleep-related breathing disorders, atherosclerotic cardiovascular disease, heart failure, chronic kidney disease, metabolic liver disease, osteoarthritis, and complex multimorbidity (2–7). Evidence from population-based studies indicates a worldwide rise in obesity (8), with Polish research documenting substantial prevalence and cardiometabolic comorbidity among working adults (9). These findings underscore the importance of understanding national trends in obesity and related conditions for public health strategies.

Routinely collected administrative data are valuable for this purpose, encompassing millions of records over several years. Nevertheless, the size of these datasets alone does not ensure the availability of the necessary epidemiological insights. Estimating prevalence or incidence from claims data or electronic health records requires clear alignment of the case definition, eligible population, observation window, look-back period, and time at risk; methodological choices can substantially influence both the absolute figures and the apparent temporal trends (10). An illustrative example is provided by obesity codes. Validation studies indicate that an obesity code may be indicative when present, yet numerous individuals meeting anthropometric criteria do not receive a corresponding clinical code in administrative records (11–15).

A diagnostic code is generated through a sequence of events: a person interacts with the healthcare system; a clinician assesses and recognizes obesity; the diagnosis is documented; and a qualifying service is reported with E66 in the required diagnosis position. A change at any stage can alter the recorded count, even if the underlying clinical population is unchanged. Conversely, a genuine increase in obesity can also contribute to higher counts. Administrative E66 series therefore represent the joint output of disease burden and the healthcare and reporting processes that make that burden visible. In primary care settings across the United Kingdom, weight was documented for approximately one-third of adults annually, with recording associated with consultations, comorbidities, and incentivisation (16). In Dutch primary care, BMI was recorded in roughly 30% of adults, and diagnosis recording was similarly selective (17). The IMPACT-O study reported BMI availability for 4.8%−38.9% of active database subjects and overweight- or obesity-related diagnosis codes for 2.9%−50.1% across seven countries (18).

Those proportions could be estimated because patient-level BMI and diagnosis data were available for defined, harmonized active populations. The Polish NFZ open-data portal, by contrast, publishes four national series containing the ICD-10 parent code E66: longitudinal registered morbidity, first-recorded patients, annual E66 patients, and annual E66-coded services (19–22). A portal user searching for a national obesity trend might reasonably expect these series to be alternative views of a single quantity. The source audit showed otherwise: their units, entry rules, look-back periods, and temporal definitions differ. In 2025, for example, the two person-based series labeled registered morbidity and annual E66 patients differ by almost fivefold. Both are official and based on E66, but they answer different questions.

The principal research question was therefore: does selecting an official NFZ E66 series materially alter the level, temporal pattern, and public-health interpretation of recorded obesity-related activity in Poland? We examined three related questions: how the unit of analysis and temporal construction determine each estimand; whether the series respond differently during a major healthcare-system shock; and which conclusions about clinical obesity remain supported or unsupported. Estimand heterogeneity in claims research is well established (10), but its practical consequences are not apparent from the headline labels on the NFZ portal. The contribution of this study is a resource-level, reproducible demonstration that selecting an official series is not merely a technical extraction decision: it changes the quantity reported and the trajectory observed.

The objectives were to: (i) define the four estimands and document their source lineage; (ii) quantify their raw and population-scaled trajectories for 2015–2025; (iii) compare their descriptive responses in 2020, the first year of the COVID-19 pandemic, without claiming a causal interrupted time-series effect; (iv) assess the influence of the 2025 endpoint; (v) contextualize the 2025 indicators using independent BMI sources without treating them as a common validation cohort; and (vi) identify the minimum data architecture required for clinical obesity and multimorbidity surveillance.

## Materials and methods

### Study design

We conducted a retrospective, descriptive ecological time-series analysis using nationally aggregated, routinely collected data. The analytical units were annual, source-specific counts of persons or services. The study tested construction-based expectations rather than a causal clinical hypothesis: a longitudinal measure that retains earlier records should be less responsive to a short-term disruption of current-year care than measures that require qualifying activity in that year. Because patient-level records and cross-source linkages were unavailable, the study could not analyse patient trajectories, overlap, clinical onset, multimorbidity sequences, treatment pathways, diagnostic sensitivity, or causal mechanisms. Reporting conformed to the RECORD statement for studies using routinely collected health data (23).

### NFZ data sources and source audit

Four publicly available datasets from the National Fund for Health (NFZ) were analyzed for the period 2015–2025 (19–22). Dataset 2,577 covers patients alive in a specified reference year who had a service recorded with a principal three-character ICD-10 code in that year or a preceding year. Dataset 2,561 includes patients whose first recorded service with a principal three-character code occurred in the NFZ data during the reference year. Dataset 2,557 comprises annual patient data with a principal diagnosis within resources, coded to five characters using ICD-10. Dataset 2,559 reports annual services with a principal diagnosis in the corresponding resource, also coded to up to five characters.

For each dataset and year, we selected the single published entry with the ICD-10 parent code E66. E66 was required to be the principal diagnosis as specified by each resource. The resulting series therefore capture a selected subset of obesity-related activity and exclude encounters in which obesity was recorded only as a secondary diagnosis. No value was imputed. The E66 parent entries contained exact counts, so small-count masking did not affect the analyzed figures. Source files were derived from SWIAD reports submitted by healthcare providers.

The final source audit enumerated all 44 annual resources (four datasets × 11 years) and recorded dataset and resource identifiers, file URLs, schema, modification dates, row counts, exact E66 values, and SHA-256 hashes in the machine-readable source manifest and Supplementary Table S1. All 44 files contained exactly one unmasked E66 parent row, with the year matching the resource metadata. The four 2025 resources were published on 2 July 2026; we therefore used 2015–2025 as the main period and 2015–2024 as an endpoint sensitivity.

### Population denominator

Each annual administrative count was scaled to the total resident population of Poland as of 30 June, using Statistics Poland (GUS) data, and expressed per 100,000 residents (24). This scaling accounts for changes in population size but not in age or sex structure, and it does not convert the numerators into prevalence or incidence measures. GUS national totals were rounded to the nearest thousand; rates were therefore reported to one decimal place. The population declined from 38.455 million in 2015 to 37.402 million in 2025. The total number of NFZ-insured persons was also considered as a potential denominator (25).

However, this measure was not used, as it provides a snapshot of administrative status at a single point in time, excludes certain non-insurance entitlement bases, and does not align with full-year numerators, whose qualifying populations and age distributions remain undisclosed. Consequently, the resident population was selected as a transparent scaling denominator rather than a population-at-risk denominator.

### Cross-sectional contextual triangulation

Two independent sources from 2025 provided context for the NFZ indicators. The precise CeZ/NFZ Zdrowe Dane workbook, BMI_250923.xlsx (SHA-256 fa6f5fd644c5eb1dd18648127db6a6955baaef129ae1f16e413480fc3ef6d754), included adults aged 18 years and over with a PESEL identifier and at least one primary-care (POZ) record reporting weight and height between January and July 2025. Values outside the ranges of 12–300 kg for weight and 100–250 cm for height were excluded. Where multiple records existed, weight and height were averaged separately before calculating BMI. The data were aggregated into nine mutually exclusive BMI categories across 380 powiats, and national totals were independently reproduced as published (26).

The NIZP PZH Health and Risk Factors Survey was undertaken in February 2025 in a nationally representative sample of 3,000 Polish residents aged 20 years or older (27, 28). BMI was calculated from self-reported weight and height, with obesity estimated at 13.9%. The triangulation compared source-specific levels within 2025; it did not treat the sources as repeated measures of one population quantity. No external dataset shared the NFZ population, observation period, age range, case definition, or denominator. We therefore did not calculate an E66-to-BMI ratio, coding sensitivity, under-capture fraction, correlation, or statistical difference between sources.

### Endpoints and statistical analysis

Primary descriptive endpoints comprised annual counts and population-adjusted administrative rates, together with absolute and percentage changes from 2015 to 2025. Additional metrics included the compound annual growth rate (CAGR), indexed values with 2015 as the baseline set at 100, year-on-year percentage changes, and services per annual E66 patient. This ratio delineates the relationship between independently published aggregates and is not indicative of population rates, patient-level probabilities, overlap measures, or diagnostic completeness estimates. For each series of counts and population-adjusted rates, an ordinary least-squares log-linear model was fitted, with the natural logarithm of the annual value as the dependent variable and calendar year as the independent variable. The annual percentage change (APC) was derived as 100 times [exp(β) – 1].

The 95% confidence intervals quantify uncertainty around the fitted log-linear pattern, not sampling variability in the complete published aggregates. A model with a one-year indicator for 2020 summarized the percentage deviation from the fitted long-term trend. The indicator was specified in advance as a descriptive pandemic-era stress test of the four data constructions and was not interpreted as a causal interrupted time-series effect (29). Sensitivity analyses excluded 2020 and limited the endpoint to 2024. Mann–Kendall tests and Theil–Sen slopes were retained as supplementary robustness measures. With only eleven observations and non-exchangeable estimands exposed to shared calendar shocks, no pooled cross-series slope or pulse comparison was performed.

Analyses were conducted using Python 3.12 and the libraries NumPy, pandas, SciPy, and Matplotlib. No random seed was deemed necessary. The Supplementary material includes the executable analysis script, all 44 source files, the source manifest, derived CSV outputs, contextual source files, and high-resolution figures.

### Ethics statement

Only publicly accessible aggregated data, devoid of direct personal identifiers, were analyzed. No individual clinical records or sensitive patient-level information were processed.

## Results

### Source definitions and availability

The source audit identified four distinct estimands (Table 1). Registered morbidity is a longitudinal record of morbidity conditioned on survival in the reference year; first-recorded patients mark entry into the observable NFZ record; annual E66 patients are those with qualifying current-year activity; and E66-coded services are events. The practical consequence is substantial: in 2025, registered morbidity included 1,534,022 persons, whereas the annual E66 patient series included 325,769—a 4.7-fold difference between two official person-based E66 indicators. Neither value is an alternative estimate of the other. All four datasets provided complete 2015–2025 series without subcode aggregation or imputation.

**Table 1:** Definitions, units, time structures and estimands of the four NFZ E66 administrative series.

Series	Dataset; 2025 resource	Unit	Published construction	Estimand used	Denominator in file
Registered morbidity	2,577; 2,187,867	Persons	Alive in reference year; principal 3-character E66 in that or an earlier year	Longitudinal administrative history	No
First-recorded patients	2,561; 2,187,862	Persons	First NFZ service with principal 3-character E66 in reference year	First administrative record	No
Annual E66 patients	2,557; 2,187,860	Persons	Qualifying principal E66 activity within year; codes to 5 characters	Annual coded patient activity	No
E66-coded services	2,559; 2,187,875	Services	Qualifying principal E66-coded services within year; repeats possible	Annual coded event volume	Not a patient denominator

For every year, the directly published E66 parent row was used; E66.0–E66.9 rows were not summed. All resources required E66 as the principal diagnosis. None of the four constructs is clinical prevalence or incidence. Algorithmic patient-deduplication details are not disclosed in the public metadata.

### Raw counts and population-scaled rates

Registered morbidity rose from 551,550 in 2015 to 1,534,022 in 2025, a 178.1 per cent increase. The number of first-recorded patients grew from 67,457 to 222,279, a 229.5 per cent increase; annual E66 patients expanded from 82,164 to 325,769, a 296.5 per cent rise; and E66-coded services rose from 131,912 to 570,543, a 332.5 per cent increase. Corresponding population-scaled rates rose from 1,434.3 to 4,101.4, from 175.4 to 594.3, from 213.7 to 871.0, and from 343.0 to 1,525.4 per 100,000 residents, respectively (see Table 2). All four series increased between 2024 and 2025, though the magnitude varied markedly by estimand. Registered morbidity rose from 1,323,563 to 1,534,022 (+15.9 per cent), first-recorded patients from 148,296 to 222,279 (+49.9 per cent), annual E66 patients from 222,674 to 325,769 (+46.3 per cent), and E66-coded services from 384,779 to 570,543 (+48.3 per cent).

**Table 2:** Annual NFZ E66 counts and population-scaled administrative rates, 2015–2025.

Year	Registered morbidity	First-recorded patients	Annual E66 patients	E66-coded services
2015	551,550 (1,434.3)	67,457 (175.4)	82,164 (213.7)	131,912 (343.0)
2016	619,979 (1,613.4)	72,638 (189.0)	91,130 (237.2)	143,861 (374.4)
2017	686,456 (1,786.6)	71,380 (185.8)	95,158 (247.7)	150,217 (391.0)
2018	754,695 (1,964.7)	73,801 (192.1)	100,477 (261.6)	161,131 (419.5)
2019	831,024 (2,164.9)	82,788 (215.7)	114,765 (299.0)	181,231 (472.1)
2020	877,794 (2,288.7)	55,943 (145.9)	83,665 (218.1)	129,649 (338.0)
2021	958,401 (2,511.4)	92,026 (241.1)	126,282 (330.9)	198,256 (519.5)
2022	1,061,909 (2,807.3)	112,766 (298.1)	158,956 (420.2)	254,251 (672.1)
2023	1,185,693 (3,145.2)	133,333 (353.7)	196,215 (520.5)	326,060 (864.9)
2024	1,323,563 (3,523.6)	148,296 (394.8)	222,674 (592.8)	384,779 (1,024.4)
2025	1,534,022 (4,101.4)	222,279 (594.3)	325,769 (871.0)	570,543 (1,525.4)

Each cell displays the count (administrative rate per 100,000 residents). Mid-year GUS population totals were reported to the nearest thousand. Patient and service numerators have different units and should not be summed or treated as interchangeable.

The 2025 increase was therefore much smaller in the longitudinal registered-morbidity construct than in the three measures that require current-year activity. These are observed values, published in resources on 2 July 2026, not projections. The mechanism cannot be determined from the released aggregates. Indexing compares the magnitude and timing of relative change while preserving the distinction between estimands (Figure 1).

**Figure 1:**
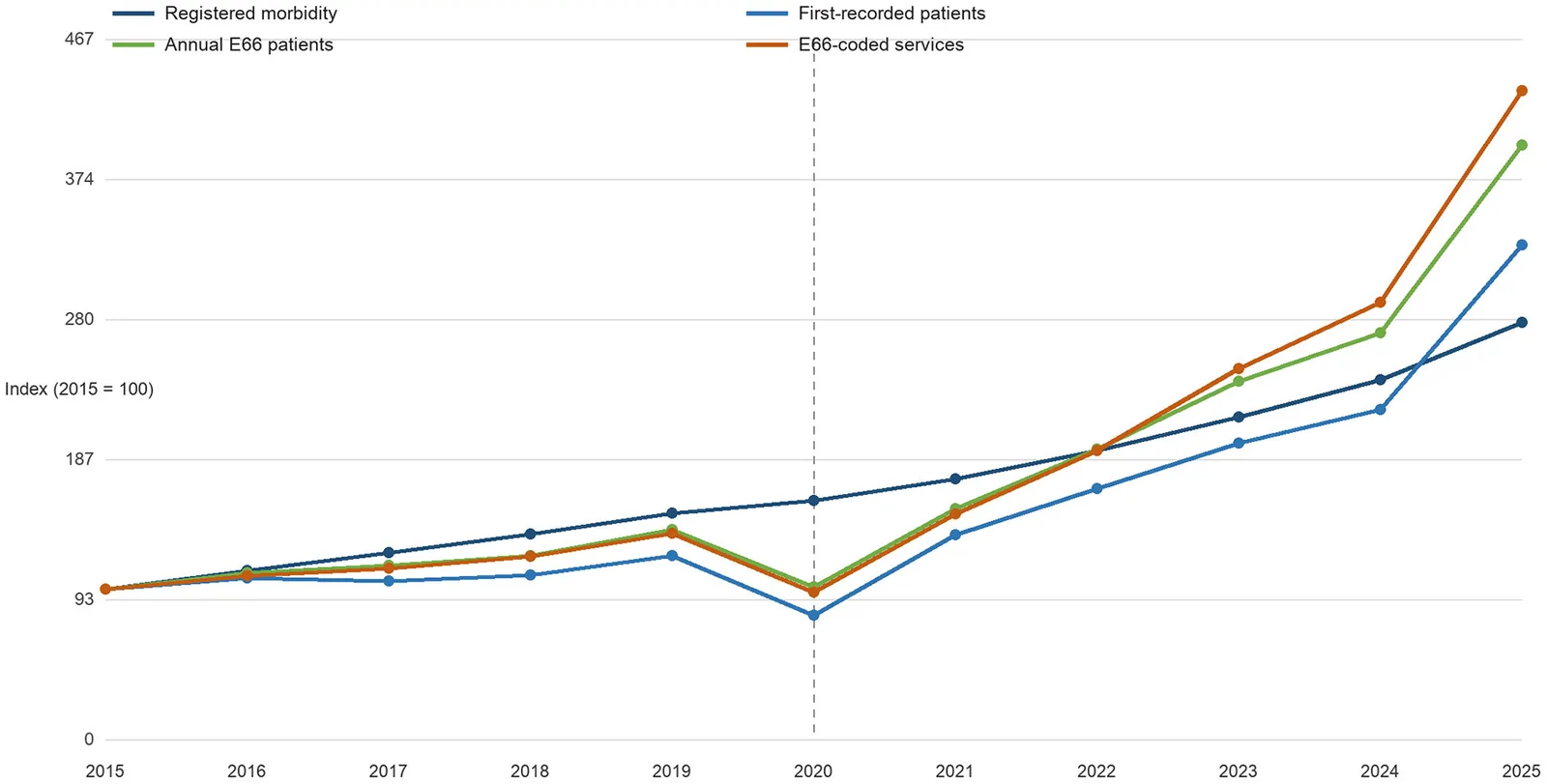
Indexed trajectories of four NFZ E66 administrative series from 2015 to 2025. Each series is indexed to its respective value in 2015 (2015 = 100). The dashed line denotes the year 2020, marking the onset of the COVID-19 pandemic. The indexing illustrates relative changes but does not imply that the constructs or units are interchangeable.

### Trend estimates and sensitivity analyses

The raw-count APC estimates were 10.1% for registered morbidity, 11.2% for first-recorded patients, 13.2% for annual E66 patients, and 14.2% for E66-coded services. Population-scaled APCs were 10.5%, 11.5%, 13.5%, and 14.5%, respectively (see Table 3). Population scaling increased APC estimates by approximately 0.3 percentage points per year but did not materially alter their direction or ranking. In the sensitivity analysis extending through 2024, all estimates remained positive (raw-count APC 8.8%−11.7%; population-scaled APC 9.1%−12.0%). Excluding 2020 did not change the overall direction of any estimate.

**Table 3:** Trend estimates for raw counts and population-scaled administrative rates, 2015–2025.

Series	Change, %	CAGR, %	APC, % (95% CI)	2020 pulse, % (95% CI); *p*
Raw counts				
Registered morbidity	178.1	10.8	10.1 (9.5–10.8)	−2.8 (−8.9 to 3.7); 0.340
First-recorded patients	229.5	12.7	11.2 (5.8–16.8)	−43.9 (−60.3 to −20.8); 0.005
Annual E66 patients	296.5	14.8	13.2 (8.4–18.2)	−39.0 (−55.7 to −16.0); 0.007
E66-coded services	332.5	15.8	14.2 (8.7–19.9)	−41.8 (−60.1 to −15.1); 0.011
Population-scaled rates				
Registered morbidity	186.0	11.1	10.5 (9.8–11.1)	−3.5 (−9.7 to 3.2); 0.253
First-recorded patients	238.8	13.0	11.5 (6.1–17.2)	−44.3 (−60.9 to −20.8); 0.005
Annual E66 patients	307.6	15.1	13.5 (8.7–18.6)	−39.4 (−56.3 to −16.1); 0.008
E66-coded services	344.7	16.1	14.5 (9.0–20.3)	−42.2 (−60.6 to −15.2); 0.011

APC, annual percentage change; CAGR, compound annual growth rate. Confidence intervals describe fitted-model uncertainty. The 2020 pulse is descriptive. Mann–Kendall, Theil–Sen and sensitivity estimates are reported in Supplementary Tables S2, S3.

The population-scaled series are shown on separate axes in Figure 2 because they represent different constructs and levels. In 2020, the first year of the COVID-19 pandemic, the estimated deviation from the fitted trend was −3.5% for registered morbidity (95% CI −9.7 to 3.2), compared with −44.3% for first-recorded patients (−60.9 to −20.8), −39.4% for annual E66 patients (−56.3 to −16.1), and −42.2% for E66-coded services (−60.6 to −15.2). The aggregate design did not support a formal test of differences between pulses. The ratio of services per annual E66 patient increased from 1.605 in 2015 to 1.751 in 2025 and was 1.550 in 2020 (Figure 3). This ratio relates two independently published aggregates; it is not a patient-level measure of contact intensity, transition, or diagnostic capture.

**Figure 2:**
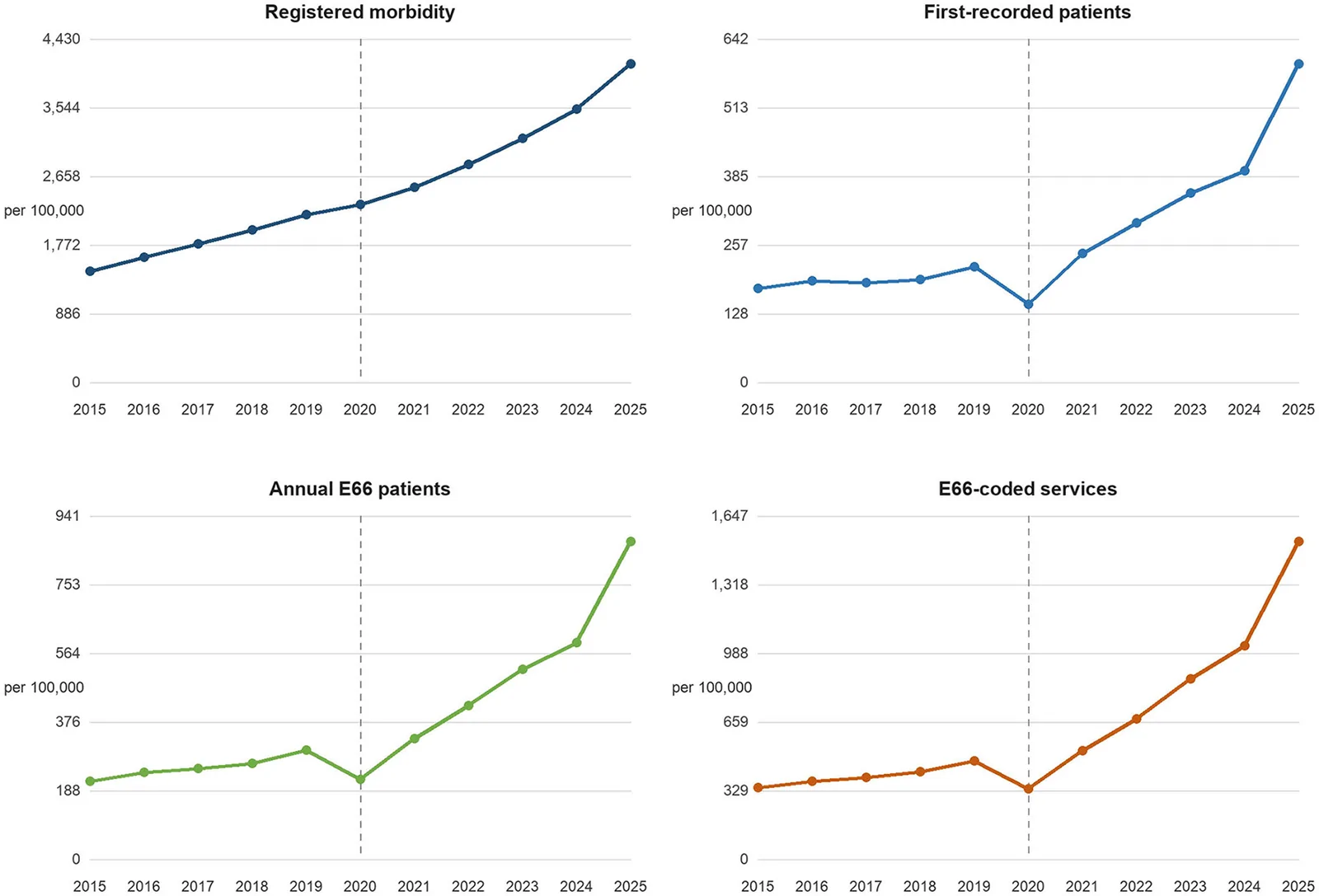
Population-adjusted administrative rates for four NFZ E66 constructs, 2015–2025. Each panel features its own y-axis and presents the annual count per 100,000 mid-year residents. The dashed line marks the year 2020, corresponding to the onset of the COVID-19 pandemic. Scaling reflects population size but does not generate prevalence or incidence rates.

**Figure 3:**
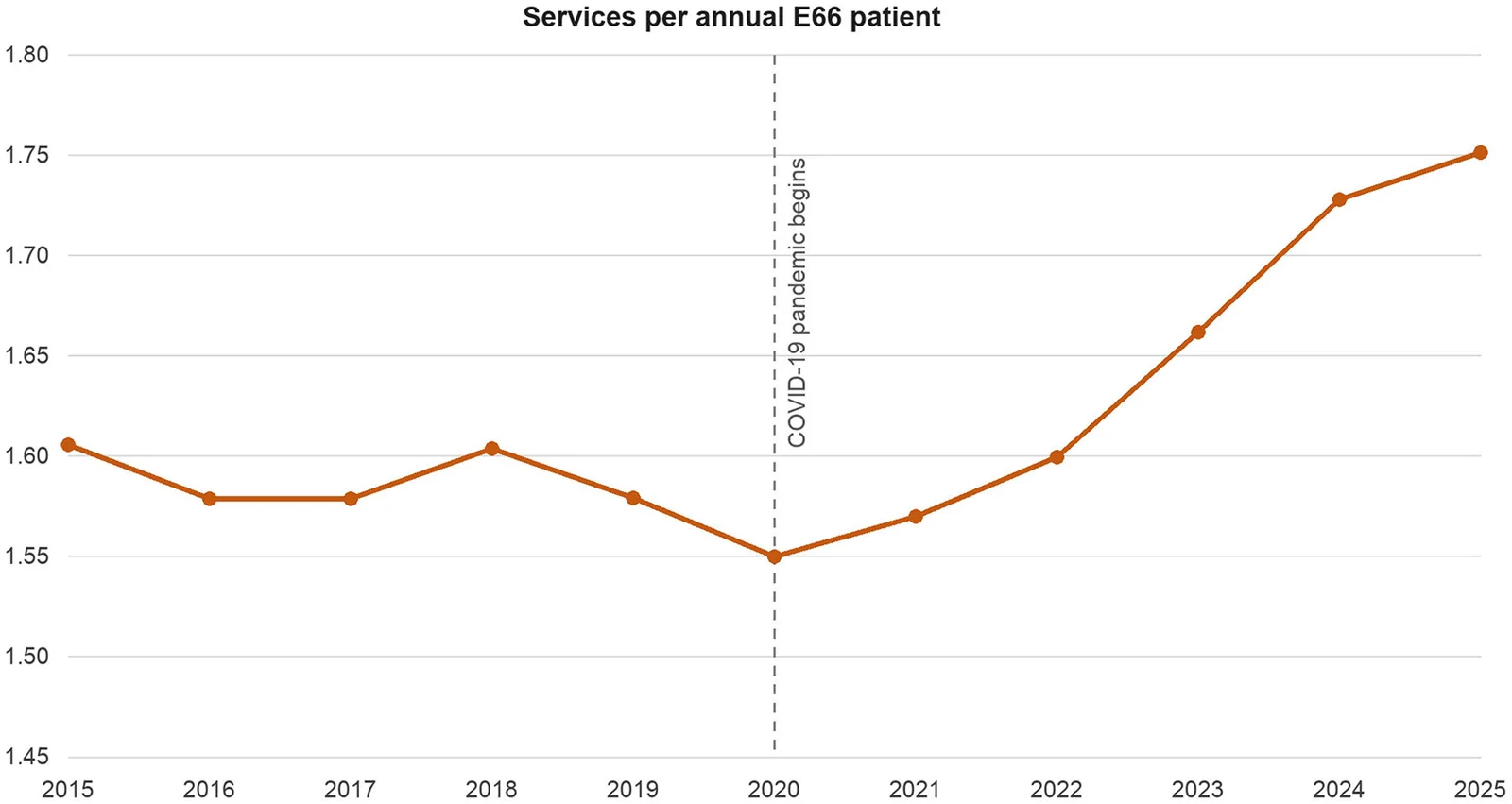
E66-coded services per annual E66 patient, 2015–2025. The ratio quantifies the aggregate coded service volume in relation to the individuals represented in the independently published annual patient series. The dashed line denotes the year 2020. This metric does not represent a patient-level contact measure, transition probability, or diagnostic capture estimate.

### Cross-sectional context in 2025

The four NFZ indicators for 2025 were 1,534,022 registered-morbidity patients, 222,279 first-recorded cases, 325,769 annual E66 patients, and 570,543 E66-coded services. The POZ BMI workbook recorded 13,402,601 adults aged 18 years or older with reportable BMI between January and July 2025; of these, 3,595,730 (26.83%) had a BMI of at least 30 kg/m^2^, while 8,646,885 (64.52%) were classified as overweight or obese. The NIZP PZH survey estimated obesity prevalence at 13.9% among residents aged 20 years or older. Although the CeZ/POZ percentage exceeded the PZH estimate, these figures do not pertain to identical populations. The CeZ source comprises adults already engaged with primary care who had reportable height and weight data during a 7-month period—a care-selected denominator that may be enriched for morbidity and clinical monitoring.

The estimate from PZH is based on a survey of randomly selected addresses and relies on self-reported height and weight, a method that typically underestimates BMI and the prevalence of obesity (30). Variations in selection criteria, age ranges, observation periods, missing data, and measurement procedures may all account for the observed discrepancies. The comparison is therefore contextual and does not assess E66 sensitivity, diagnostic detection, or population under-coding (see Table 4).

**Table 4:** Cross-sectional contextual comparison of NFZ E66, POZ-reported BMI and NIZP PZH sources in 2025.

Source	Construct	Population/window	Denominator	Result	Key limit
NFZ registered morbidity	Living persons with current/earlier principal E66 record	Age not stated; 2025	GUS resident population for scaling	1,534,022; 4,101.4/100,000	Longitudinal history; not prevalence
NFZ first-recorded	First principal E66 record in NFZ	Age not stated; 2025	GUS resident population for scaling	222,279; 594.3/100,000	Not clinical incidence
NFZ annual E66 patients	Persons with principal E66-coded activity	Age not stated; 2025	GUS resident population for scaling	325,769; 871.0/100,000	Annual coded activity
NFZ E66-coded services	Principal E66-coded services	Services; 2025	GUS resident population for scaling	570,543; 1,525.4/100,000	Events, not persons
CeZ/NFZ POZ BMI	BMI ≥30 kg/m^2^ from reported weight and height	Adults 18+ with reportable BMI; Jan–Jul 2025	13,402,601 source-eligible adults	3,595,730; 26.83%	Selected care population; missingness unknown
NIZP PZH survey	BMI ≥30 kg/m^2^ from self-report	Representative survey, age 20+; Feb 2025	Representative sample, *n* = 3,000	13.9%	Different sampling and measurement

Values are source-specific and are presented for cross-sectional contextual triangulation, not as estimates of one population quantity. Differences may reflect selection, age range, observation window, missingness, measurement method, diagnosis recording, and true disease burden; these contributions cannot be separated.

## Discussion

### Principal findings

The choice of an NFZ series altered both the magnitude and the temporal behavior of the resulting E66 indicator. In 2025, two official person-based series differed by 4.7-fold, and endpoint growth across all four series ranged from 178.1 to 332.5%. These are not minor variations around a single national obesity measure; they reflect differences in how longitudinal recorded history, entry into the record, current-year patient activity, or service events are measured. The empirical contribution is therefore not merely that claims-based estimands depend on definitions. It is the demonstration that a portal user can select the same parent diagnosis code from the same national open-data environment and obtain substantively different, internally valid answers because the underlying research questions differ.

All four series increased over 2015–2025. That common direction is consistent with a genuine rise in clinical obesity, but it may also reflect greater recognition, shifts in diagnostic positioning, changes in access or care-seeking, more intensive obesity-focused care, and changes in reporting. The available aggregates capture the combined output of these processes and cannot partition their contributions. Population scaling addresses only changes in population size; it does not resolve the numerator or account for age and sex structure. The appropriate conclusion is therefore positive but bounded: documented principal-E66-coded activity increased, potentially in part because the underlying burden of obesity increased, while the magnitude of clinical prevalence or incidence remains unidentified.

### Data architecture as an empirical stress test

The contrasting 2020 patterns provide a pandemic-era stress test for the four constructions. The first year of the COVID-19 pandemic was associated with broad reductions in the use of publicly financed healthcare in Poland; a national analysis found declines across most areas of care, including an almost 18% reduction in primary-care physician consultations (31). Against that documented system-wide disruption, the observed E66 pattern is consistent with the temporal definitions: the longitudinal registered-morbidity construct retained people with earlier qualifying records and showed only a modest, imprecise deviation, whereas the three series requiring current-year activity showed deviations of approximately −39 to −44%. The analysis does not identify a causal pandemic effect or a specific mechanism, but it demonstrates that data architecture determines how an administrative indicator responds to a major healthcare-system shock.

The 2025 pattern provides a second, differently directed stress test: registered morbidity increased by 15.9%, while the three current-year activity measures rose by 46.3%−49.9%. Several mechanisms are plausible. KOS-BAR, introduced in 2021 and operational from 2022, was amended in June 2024 and remained in effect through 2025 (32, 33); it may have increased structured obesity-related activity in a selected population. Changes in POZ activity or reporting, greater clinical attention to obesity, altered diagnostic positioning, and broader use of incretin-based pharmacotherapy could also have increased opportunities to record E66. A genuine increase in clinical obesity may have contributed as well. Because the aggregates do not link diagnoses to prescriptions, identify care settings, include secondary diagnoses, or document changes in deduplication and SWIAD processing, these explanations remain hypotheses rather than findings.

Distinguishing these mechanisms requires patient-level records stratified by care setting and diagnosis position, linkage to prescriptions and repeated anthropometry, versioned reporting specifications, and processing logs. The code is only one component of the measurement system. The unit of analysis, eligible population, observation window, look-back period, survival condition, diagnosis position, deduplication, and linkage jointly determine the estimand. The central practical lesson is that choosing a dataset is equivalent to choosing the public-health question being addressed.

### European evidence and what richer data permit

European studies illustrate the distinction between administrative indicators based on diagnosis and clinical obesity. In Germany, Steffen and colleagues delineated annual diagnosed obesity using E66 alongside a confirmed diagnosis criterion, dividing cases by the mid-year statutory-insured population. Their findings showed an increase in diagnosed prevalence from 9.2% in 2009 to 11.2% in 2018, enabling analysis by age, sex, and region (34). This approach used an explicitly defined eligible denominator and stratification variables absent from the Polish open E66 datasets. Nonetheless, comparison with survey data remained imperative, as diagnosed prevalence did not fully correspond with anthropometric prevalence.

The Danish validation study explains why a code may be considered reliable when present, despite its limited comprehensiveness for population surveillance (14). Additional evidence from studies in the United Kingdom and the Netherlands indicates that BMI measurements and diagnostic recording are selective processes shaped by contact patterns and comorbidities (16, 17). IMPACT-O is the most proximate multi-country example demonstrating the data architecture needed to address the initial research question: an active population denominator, patient-level BMI and diagnoses, longitudinal observation, and a common data model that enables country-specific estimates of recording practices and obesity-related complications (18). The authors nevertheless advise caution, noting that variability in database coverage and care settings across countries limits the comparability of interpretations. These studies do not validate the Polish aggregate data, and their percentages should not be applied directly to Poland.

They demonstrate why certain questions are answerable elsewhere: the relevant numerator and denominator are present within the same eligible population, with linkage and covariates. Our failure to estimate trajectories of clinical obesity or multimorbidity is thus not attributable to an insufficient sample size; rather, it is constrained by the structure of the data made available.

### Minimum data architecture for obesity surveillance

The analytical target should be defined before an NFZ series is selected. Table 5 maps common public-health questions to the numerators, denominators, time structures, and linkages they require. No single E66 series is universally preferable: registered morbidity can track the cumulative recorded history among survivors; annual E66 patients can track current-year patient activity; and E66-coded services can track event volume. None alone provides clinical prevalence, onset, recording completeness, or attributable economic burden.

**Table 5:** Minimum data architecture for national obesity and multimorbidity surveillance.

Analytical target	Required numerator	Required denominator/time	Required linkage/variables	Missing from current aggregates
Population prevalence	Validated obesity definition; measurement date	Defined resident/eligible population at same time	Age, sex, anthropometry; missing BMI	Aligned case denominator and anthropometry
Clinical incidence/onset	New clinical obesity meeting prespecified criteria	Disease-free cohort; adequate look-back and time at risk	Longitudinal linkage; repeated measurements	Disease-free status, onset date, person-time
Administrative first record	First qualifying E66 record	Continuously observable administrative cohort	Documented look-back and enrolment	Continuous eligibility and complete history
Care trajectory	Ordered contacts, diagnoses, procedures and treatment	Linked patient cohort over time	Dates, settings, providers, repeated events	Patient linkage and sequence
Obesity multimorbidity	Obesity plus validated comorbidity definitions	Common linked cohort	Diagnosis timing, severity, BMI, medication, outcomes	Co-occurrence and temporal ordering
Diagnosis recording completeness	E66 code	Patients with independently ascertained obesity in same database	Linked code and anthropometry; prespecified window	Common validation population
Resource use/economics	Obesity-attributable services and costs	Defined eligible cohort and comparator	Patient linkage, costs, attribution method, confounding	Cost linkage and attribution

### Implications for public health and open-data practice

For researchers and policy analysts, each NFZ estimate should be reported with its dataset and resource identifier, analytical unit, diagnosis position, temporal structure, extraction rule, numerator, denominator, and scale. Labels such as “number of patients with obesity” are insufficient when different official resources produce person counts that differ almost fivefold. Population-scaled indicators can monitor documented activity and generate hypotheses for service planning, provided their estimands are explicit. For data providers, high-value improvements include stable annual denominators, age- and sex-stratified outputs, documented deduplication and look-back rules, care-setting and diagnosis-position flags, and privacy-preserving linkage between E66, repeated BMI measurements, comorbidities, treatments, and costs. Such infrastructure would support the clinical and public-health questions that motivated this audit but cannot be answered using the currently released aggregates.

The broader implication extends beyond obesity. National open-data systems often organize outputs around familiar diagnostic labels, while the rules that generate each quantity remain scattered across metadata, schemas, and annual resources. An explicit estimand statement—unit, eligible population, diagnostic position, look-back period, deduplication rule, observation window, and denominator—would make those technical details visible properties of the indicator. This is necessary not only for reproducibility but also to prevent different administrative quantities from becoming competing public narratives about the same disease.

### Strengths and limitations

Strengths include a reproducible audit of four official source constructions, extraction of all 44 annual resources, direct selection of E66 parent rows, verification of resource metadata and checksums, and a single transparent analysis pipeline. The 2020 pandemic-era contrast served as an externally contextualized stress test of the constructions, and external BMI sources were used without treating unmatched populations as validation cohorts. The principal limitation is ecological aggregation: patient overlap, clinical severity, BMI, age, sex, treatment, cost, and temporal sequence were unavailable. Only principal diagnoses were included, so the series exclude activity in which obesity was recorded solely as a secondary diagnosis and may be sensitive to changes in diagnosis positioning. Public metadata do not disclose algorithmic deduplication.

The resident-population scale is not the population at risk and does not adjust for changes in age or sex structure. With eleven observations, APC confidence intervals describe fit to a log-linear model and are sensitive to functional form and individual years; they are not measures of sampling uncertainty. The 2020 indicator remains descriptive, and the pandemic's causal contribution cannot be estimated. The mechanisms underlying the 2025 increase cannot be determined from the released aggregates. Selection, missingness, age range, observation window, and measurement differences preclude formal validation against CeZ and PZH. The study was not preregistered, and the model-based analyses were exploratory.

## Conclusion

Polish NFZ open data do not provide a single national E66 trajectory but four: longitudinal recorded history, first administrative records, annual patient activity, and annual service volume. All increased between 2015 and 2025, and a genuine rise in clinical obesity may have contributed to that growth. However, the four series differed substantially in level and in their response to the pandemic year of 2020 and the abrupt increase in 2025. The aggregates cannot separate changes in disease burden from changes in recognition, access, recording, diagnosis positioning, or service delivery. Their valid use is therefore to monitor explicitly defined forms of documented E66-coded activity—not to estimate clinical prevalence or incidence.

The choice of an official series is not merely a technical extraction decision; it determines the quantity reported and the public health question addressed. National clinical obesity surveillance requires aligned denominators, anthropometry, diagnostic position, and longitudinal patient-level linkage.

## Data Availability

The original contributions presented in the study are included in the article/supplementary material, further inquiries can be directed to the corresponding author.
