# Supplementary material for: Same ICD-10 code, different estimands: four Polish National Health Fund obesity series, 2015–2025
Source: Front Public Health. 2026 Sep 18;14:1920632. doi: 10.3389/fpubh.2026.1920632 (PMC13630639; doi:10.3389/fpubh.2026.1920632)
Supplement: Supplementary file 1 [file Table_1.docx]

**Supplementary Material**

*Supplementary tables and reproducibility inventory*

All values below were verified against the archived analysis outputs on 14 August 2026. The NFZ audit required one exact, unmasked E66 parent row in every annual source file. Full URLs and 64-character SHA-256 hashes are supplied in NFZ_source_manifest.csv.

# Supplementary Table S1. Complete NFZ annual resource inventory

| **Series** | **Year** | **Dataset** | **Resource** | **Format** | **Modified** | **Exact E66 count** | **SHA-256 prefix** |
| --- | --- | --- | --- | --- | --- | --- | --- |
| Registered morbidity | 2015 | 2577 | 34792 | CSV | 2024-04-23 | 551,550 | fff12fdca872 |
| Registered morbidity | 2016 | 2577 | 34793 | CSV | 2024-04-23 | 619,979 | fef34d780638 |
| Registered morbidity | 2017 | 2577 | 34794 | CSV | 2024-04-23 | 686,456 | 35455f0e9c86 |
| Registered morbidity | 2018 | 2577 | 34795 | CSV | 2024-04-23 | 754,695 | a44f3094d8ea |
| Registered morbidity | 2019 | 2577 | 34796 | CSV | 2024-04-23 | 831,024 | 10d759c196cc |
| Registered morbidity | 2020 | 2577 | 34797 | CSV | 2024-04-23 | 877,794 | cb86034f6f13 |
| Registered morbidity | 2021 | 2577 | 43072 | CSV | 2024-04-23 | 958,401 | 09223e597a40 |
| Registered morbidity | 2022 | 2577 | 51464 | XML | 2024-04-23 | 1,061,909 | 51539dd59e19 |
| Registered morbidity | 2023 | 2577 | 65400 | XML | 2025-03-18 | 1,185,693 | 49c07b630245 |
| Registered morbidity | 2024 | 2577 | 65401 | XML | 2025-03-18 | 1,323,563 | ba5352cda02c |
| Registered morbidity | 2025 | 2577 | 2187867 | XML | 2026-07-02 | 1,534,022 | 0f688eafb1b2 |
| First recorded patients | 2015 | 2561 | 43097 | XML | 2024-04-23 | 67,457 | 2389546031f7 |
| First recorded patients | 2016 | 2561 | 43098 | XML | 2024-04-23 | 72,638 | 765409f09f5b |
| First recorded patients | 2017 | 2561 | 43099 | XML | 2024-04-23 | 71,380 | 33c2328cd521 |
| First recorded patients | 2018 | 2561 | 43100 | XML | 2024-04-23 | 73,801 | bd279efd1be0 |
| First recorded patients | 2019 | 2561 | 43101 | XML | 2024-04-23 | 82,788 | e7f8994ecad0 |
| First recorded patients | 2020 | 2561 | 43102 | XML | 2024-04-23 | 55,943 | cd24780345e3 |
| First recorded patients | 2021 | 2561 | 43103 | XML | 2024-04-23 | 92,026 | cdad5267d237 |
| First recorded patients | 2022 | 2561 | 51463 | XML | 2024-04-23 | 112,766 | 91acc753aa41 |
| First recorded patients | 2023 | 2561 | 65398 | XML | 2025-03-18 | 133,333 | 3d56814ce550 |
| First recorded patients | 2024 | 2561 | 65399 | XML | 2025-03-18 | 148,296 | b23ff3fff53a |
| First recorded patients | 2025 | 2561 | 2187862 | XML | 2026-07-02 | 222,279 | 3d10a17a0e94 |
| Annual E66 patients | 2015 | 2557 | 34681 | CSV | 2024-04-23 | 82,164 | 4a2730fc1cb7 |
| Annual E66 patients | 2016 | 2557 | 34682 | CSV | 2024-04-23 | 91,130 | 1ef8c7f84363 |
| Annual E66 patients | 2017 | 2557 | 34683 | CSV | 2024-04-23 | 95,158 | 73acb68e730a |
| Annual E66 patients | 2018 | 2557 | 34684 | CSV | 2024-04-23 | 100,477 | 896284731b9f |
| Annual E66 patients | 2019 | 2557 | 34685 | CSV | 2024-04-23 | 114,765 | 07ce4d2615a3 |
| Annual E66 patients | 2020 | 2557 | 34686 | CSV | 2024-04-23 | 83,665 | d74450965488 |
| Annual E66 patients | 2021 | 2557 | 43104 | XML | 2024-04-23 | 126,282 | 9bb53b9aa204 |
| Annual E66 patients | 2022 | 2557 | 51460 | XML | 2024-04-23 | 158,956 | 3423bce0b4ea |
| Annual E66 patients | 2023 | 2557 | 65396 | XML | 2025-03-18 | 196,215 | 68be5c5142f6 |
| Annual E66 patients | 2024 | 2557 | 65397 | XML | 2025-03-18 | 222,674 | 98029d3a1084 |
| Annual E66 patients | 2025 | 2557 | 2187860 | XML | 2026-07-02 | 325,769 | e9c3f3647ac2 |
| E66-coded services | 2015 | 2559 | 43080 | XML | 2024-04-23 | 131,912 | dddbc494145f |
| E66-coded services | 2016 | 2559 | 43081 | XML | 2024-04-23 | 143,861 | 0d883ffdcf40 |
| E66-coded services | 2017 | 2559 | 43082 | XML | 2024-04-23 | 150,217 | 4f41c43f64e2 |
| E66-coded services | 2018 | 2559 | 43083 | XML | 2024-04-23 | 161,131 | 3eef72d11a77 |
| E66-coded services | 2019 | 2559 | 43084 | XML | 2024-04-23 | 181,231 | 2eee732cee84 |
| E66-coded services | 2020 | 2559 | 43087 | XML | 2024-04-23 | 129,649 | a040c9f26519 |
| E66-coded services | 2021 | 2559 | 43089 | XML | 2024-04-23 | 198,256 | 2114e891e9cf |
| E66-coded services | 2022 | 2559 | 51462 | XML | 2024-04-23 | 254,251 | f41cd8386a3c |
| E66-coded services | 2023 | 2559 | 65404 | XML | 2025-03-18 | 326,060 | 4df5ae37228b |
| E66-coded services | 2024 | 2559 | 65405 | XML | 2025-03-18 | 384,779 | cb95ea0bae27 |
| E66-coded services | 2025 | 2559 | 2187875 | XML | 2026-07-02 | 570,543 | 7bc64e236c33 |

Note: Full titles, file/API URLs, source row counts, headers, file sizes, access date, and complete SHA-256 hashes are in NFZ_source_manifest.csv. Every resource contained exactly one E66 parent row.

# Supplementary Table S2. Individual-series trend and 2020 pulse summaries

| **Series** | **Scale** | **Log-linear APC, % (95% CI)** | **Mann–Kendall τ; p** | **Theil–Sen APC, % (95% CI)** | **2020 pulse, % (95% CI)** |
| --- | --- | --- | --- | --- | --- |
| Registered morbidity | Raw count | 10.1 (9.5 to 10.8) | 1.000; <0.001 | 10.0 (9.5 to 10.8) | -2.8 (-8.9 to 3.7) |
| Registered morbidity | Per 100,000 | 10.5 (9.8 to 11.1) | 1.000; <0.001 | 10.3 (9.8 to 11.2) | -3.5 (-9.7 to 3.2) |
| First recorded patients | Raw count | 11.2 (5.8 to 16.8) | 0.782; <0.001 | 11.0 (5.4 to 17.1) | -43.9 (-60.3 to -20.8) |
| First recorded patients | Per 100,000 | 11.5 (6.1 to 17.2) | 0.782; <0.001 | 11.4 (5.7 to 17.5) | -44.3 (-60.9 to -20.8) |
| Annual E66 patients | Raw count | 13.2 (8.4 to 18.2) | 0.855; <0.001 | 12.2 (7.9 to 18.4) | -39.0 (-55.7 to -16.0) |
| Annual E66 patients | Per 100,000 | 13.5 (8.7 to 18.6) | 0.855; <0.001 | 12.6 (8.0 to 18.8) | -39.4 (-56.3 to -16.1) |
| E66-coded services | Raw count | 14.2 (8.7 to 19.9) | 0.818; <0.001 | 12.6 (7.3 to 21.1) | -41.8 (-60.1 to -15.1) |
| E66-coded services | Per 100,000 | 14.5 (9.0 to 20.3) | 0.818; <0.001 | 12.9 (7.4 to 21.6) | -42.2 (-60.6 to -15.2) |

APC, annual percentage change. Pulse estimates are descriptive deviations from a log-linear trend and are not causal interrupted-time-series effects. No pooled cross-series slope test was used.

# Supplementary Table S3. Endpoint and 2020-exclusion sensitivities

| **Series** | **Scale** | **Sensitivity** | **APC, % (95% CI)** | **p** | **n years** |
| --- | --- | --- | --- | --- | --- |
| Registered morbidity | Raw count | 2015–2024 endpoint | 9.8 (9.2 to 10.3) | <0.001 | 10 |
| Registered morbidity | Raw count | 2015–2025 excluding 2020 | 10.1 (9.5 to 10.8) | <0.001 | 10 |
| Registered morbidity | Per 100,000 | 2015–2024 endpoint | 10.1 (9.5 to 10.6) | <0.001 | 10 |
| Registered morbidity | Per 100,000 | 2015–2025 excluding 2020 | 10.5 (9.8 to 11.1) | <0.001 | 10 |
| First recorded patients | Raw count | 2015–2024 endpoint | 8.8 (3.5 to 14.4) | 0.005 | 10 |
| First recorded patients | Raw count | 2015–2025 excluding 2020 | 11.2 (7.7 to 14.7) | <0.001 | 10 |
| First recorded patients | Per 100,000 | 2015–2024 endpoint | 9.1 (3.7 to 14.8) | 0.004 | 10 |
| First recorded patients | Per 100,000 | 2015–2025 excluding 2020 | 11.5 (8.0 to 15.1) | <0.001 | 10 |
| Annual E66 patients | Raw count | 2015–2024 endpoint | 11.0 (6.3 to 16.0) | <0.001 | 10 |
| Annual E66 patients | Raw count | 2015–2025 excluding 2020 | 13.2 (10.0 to 16.5) | <0.001 | 10 |
| Annual E66 patients | Per 100,000 | 2015–2024 endpoint | 11.3 (6.5 to 16.4) | <0.001 | 10 |
| Annual E66 patients | Per 100,000 | 2015–2025 excluding 2020 | 13.5 (10.2 to 17.0) | <0.001 | 10 |
| E66-coded services | Raw count | 2015–2024 endpoint | 11.7 (6.3 to 17.4) | <0.001 | 10 |
| E66-coded services | Raw count | 2015–2025 excluding 2020 | 14.2 (10.3 to 18.2) | <0.001 | 10 |
| E66-coded services | Per 100,000 | 2015–2024 endpoint | 12.0 (6.5 to 17.8) | <0.001 | 10 |
| E66-coded services | Per 100,000 | 2015–2025 excluding 2020 | 14.5 (10.6 to 18.6) | <0.001 | 10 |

# Supplementary Table S4. Annual counts and population-scaled administrative rates

| **Year** | **GUS mid-year population** | **Registered morbidity, n (per 100,000)** | **First recorded patients, n (per 100,000)** | **Annual E66 patients, n (per 100,000)** | **E66-coded services, n (per 100,000)** |
| --- | --- | --- | --- | --- | --- |
| 2015 | 38,455,000 | 551,550 (1,434.3) | 67,457 (175.4) | 82,164 (213.7) | 131,912 (343.0) |
| 2016 | 38,427,000 | 619,979 (1,613.4) | 72,638 (189.0) | 91,130 (237.2) | 143,861 (374.4) |
| 2017 | 38,422,000 | 686,456 (1,786.6) | 71,380 (185.8) | 95,158 (247.7) | 150,217 (391.0) |
| 2018 | 38,413,000 | 754,695 (1,964.7) | 73,801 (192.1) | 100,477 (261.6) | 161,131 (419.5) |
| 2019 | 38,386,000 | 831,024 (2,164.9) | 82,788 (215.7) | 114,765 (299.0) | 181,231 (472.1) |
| 2020 | 38,354,000 | 877,794 (2,288.7) | 55,943 (145.9) | 83,665 (218.1) | 129,649 (338.0) |
| 2021 | 38,162,000 | 958,401 (2,511.4) | 92,026 (241.1) | 126,282 (330.9) | 198,256 (519.5) |
| 2022 | 37,827,000 | 1,061,909 (2,807.3) | 112,766 (298.1) | 158,956 (420.2) | 254,251 (672.1) |
| 2023 | 37,698,000 | 1,185,693 (3,145.2) | 133,333 (353.7) | 196,215 (520.5) | 326,060 (864.9) |
| 2024 | 37,563,000 | 1,323,563 (3,523.6) | 148,296 (394.8) | 222,674 (592.8) | 384,779 (1,024.4) |
| 2025 | 37,402,000 | 1,534,022 (4,101.4) | 222,279 (594.3) | 325,769 (871.0) | 570,543 (1,525.4) |

GUS national headline totals are reported to the nearest thousand. These are administrative rates, not prevalence or incidence estimates.

# Supplementary Table S5. Cross-series ratios

| **Year** | **Registered morbidity / annual patients** | **First recorded / annual patients** | **Services / annual patient** |
| --- | --- | --- | --- |
| 2015 | 6.713 | 0.821 | 1.605 |
| 2016 | 6.803 | 0.797 | 1.579 |
| 2017 | 7.214 | 0.750 | 1.579 |
| 2018 | 7.511 | 0.735 | 1.604 |
| 2019 | 7.241 | 0.721 | 1.579 |
| 2020 | 10.492 | 0.669 | 1.550 |
| 2021 | 7.589 | 0.729 | 1.570 |
| 2022 | 6.681 | 0.709 | 1.600 |
| 2023 | 6.043 | 0.680 | 1.662 |
| 2024 | 5.944 | 0.666 | 1.728 |
| 2025 | 4.709 | 0.682 | 1.751 |

Ratios are derived from separate national aggregates. They are not patient-level overlaps, transitions, capture fractions, or probabilities.

# Supplementary Table S6. Contextual-source and denominator audit

| **Source** | **Version** | **Population/window** | **Verified result** | **Checksum/source file** | **Analytical role/limit** |
| --- | --- | --- | --- | --- | --- |
| CeZ/NFZ BMI | BMI_250923.xlsx | Adults 18+ with PESEL and reportable POZ weight/height; Jan–Jul 2025 | 13,402,601 total; 3,595,730 BMI ≥30 (26.83%); 8,646,885 BMI ≥25 (64.52%) | fa6f5fd644c5… | Context only; selected care population; missingness unknown |
| NIZP PZH | 2025 report, Chapter 13 | Representative sample n=3,000, age 20+; random GUS-address sample; Feb 2025 | 13.9% obesity; weight and height self-reported | a6eb96ee827b… | Context only; different sampling and measurement process |
| GUS | 2015–2025 editions | National resident population as of 30 June; totals rounded to nearest 1,000 | 38.455 million (2015) to 37.402 million (2025) | See population_denominators.csv | Scaling denominator, not eligible population at risk |
